# Associations between workplace affiliation and phlebotomy practices regarding patient identification and test request handling practices in primary healthcare centres: a multilevel model approach

**DOI:** 10.1186/s12913-015-1157-9

**Published:** 2015-11-10

**Authors:** Karin Nilsson, Christina Juthberg, Johan Söderberg, Karin Bölenius, Kjell Grankvist, Christine Brulin, Marie Lindkvist

**Affiliations:** Department of Nursing, Umeå University, Umeå, Sweden; Department of Medical Biosciences, Clinical Chemistry, Umeå University, Umeå, Sweden; Department of Public Health and Clinical Medicine, Epidemiology and Global Health, Umeå University, Umeå, Sweden; Department of Statistics, Umeå University, Umeå, Sweden

**Keywords:** Clinical practice guidelines, Guideline adherence, Nursing, Phlebotomy, Professional socialization, Venous blood specimen collection, Workplace affiliation

## Abstract

**Background:**

Clinical practice guidelines aim to enhance patient safety by reducing inappropriate variations in practice. Despite considerable efforts to enhance the use of clinical practice guidelines, adherence is often suboptimal. We investigated to what extent workplace affiliation explains variation of self-reported adherence to venous blood specimen collection regarding patient identification and test request handling practices, taking into consideration other primary healthcare centre and individual phlebotomist characteristics.

**Methods:**

Data were collected through a questionnaire survey of 164 phlebotomy staff from 25 primary healthcare centres in northern Sweden. To prevent the impact of a large-scale education intervention in 2008, only baseline data, collected over a 3-month period in 2006–2007, were used and subjected to descriptive statistics and multilevel logistic analyses.

**Results:**

In two patient identification outcomes, stable high median odds ratios (MOR) were found in both the empty model, and in the adjusted full model including both individual and workplace factors. Our findings suggest that variances among phlebotomy staff can be largely explained by primary healthcare centre affiliation also when individual and workplace demographic characteristics were taken in consideration. Analyses showed phlebotomy staff at medium and large primary healthcare centres to be more likely to adhere to guidelines than staff at small centres. Furthermore, staff employed shorter time at worksite to be more likely to adhere than staff employed longer. Finally, staff performing phlebotomy every week or less were more likely to adhere than staff performing phlebotomy on a daily basis.

**Conclusion:**

Workplace affiliation largely explains variances in self-reported adherence to venous blood specimen collection guidelines for patient identification and test request handling practices among phlebotomy staff. Characteristics of the workplace, as well as of the individual phlebotomist, need to be identified in order to design strategies to improve clinical practice in this and other areas.

## Background

Clinical practice guidelines (CPG) aim to guide healthcare staff in decision-making and management of healthcare procedures in order to enhance patient safety. CPGs are usually consensus statements on best available practice/evidence-based practice (EBP) in a particular area, and are increasingly embraced by international healthcare organizations such as WHO [1] and regarded as an indispensable part of professional quality systems [2]. Successfully implemented CPGs are considered to promote cost effectiveness and quality of care and to enhance patient safety by reducing inappropriate variations in practice [3–5]. A growing body of literature examines factors influencing the use of CPGs in healthcare settings, including guideline characteristics (easily/difficult to understand), implementation strategies, staff characteristics, environmental characteristics [6], organizational aspects, occupational or individual aspects [7–10] the size of [11], as well as the structural characteristics of the workplace such as situated in rural areas or not [12]. Individual barriers influencing CPG use include e.g., lack of awareness of an existing CPG, unfamiliarity of content, lack of motivation, lack of time, lack of training, resistance to change, and lack of “local champions” [13]. Environmental barriers to CPG use are exemplified by organizational aspects such as heavy workload, limited time or personnel, and beliefs of peers and social norms [14]. The majority of studies on CPG use focus on physician behavior, whereas nurses’ use of CPGs is less studied. Nurses have been suggested to report more frequent use of, and positive attitudes towards guidelines compared to physicians [15]. However, in a recent published review on attitudes towards evidence-based practice among physicians and nurses, both professions were believed to welcome EBP, since EBP was considered to improve patient care [16]. Adherence to CPG among nurses has been shown to vary between different units [17], a result which is in line with the findings in a study revealing significant variations between, but not within, units [18].

Venous blood specimen collection (VBSC) is a common procedure within health care facilities. The use of clinical laboratory test results in diagnostic decision making or treatment evaluation is an essential part of clinical medicine [19]. The “laboratory testing cycle” or “total testing procedure” consists of several steps between the clinician ordering a laboratory test, the blood been drawn from a patient’s vein, and the test result returned to the clinician [19, 20]. Reliable evidence demonstrates that the vast majority of laboratory errors occur in the pre-analytical phase [21]. Examples of errors include improper patient identification (ID) [22], specimen mix-up [19], and miss-labelling of test tubes [23]. Hazardous consequences of patient ID errors are for example incorrect diagnosis, incorrect treatment, and failing treatment evaluation [24]. Recent studies have demonstrated varying levels of VBSC practice guideline adherence, with hospital clinical chemistry laboratory staff reporting higher levels of adherence to guidelines than hospital ward staff and primary healthcare centre (PHC) staff [25–29]. Significant variations in blood specimen hemolysis indices among PHCs also reflected the varying quality of pre-analytical procedures [30]. Despite considerable efforts to increase the use of CPGs among healthcare staff, adherence is still often suboptimal. Empirical research on the relationship between workplace affiliation and healthcare staff adherence to VBSC practice guidelines is currently lacking. We hypothesized contextual factors at different workplaces to influence VBSC guideline adherence. The aim of this study was to explore to what extent workplace affiliation explains variation of self-reported adherence to VBSC practices regarding patient ID and test request handling, taking into consideration fixed PHC workplace and individual phlebotomist characteristics.

## Methods

### Design

Data used in this cross-sectional study are part of a larger dataset collected in 2006–2011, including baseline, intervention (a large scale VBSC education program), and evaluation of intervention data. To ensure non-influenced procedures among phlebotomists, only baseline data collected in 2006–2007, prior to intervention, were used in this study.

### Measures

Dependent variables in the study were: levels of adherence to VBSC guidelines regarding patient ID, and test request handling procedures. Four items from a venous blood sampling questionnaire (VBSQ) (described below) were used to cover the outcome variables. Workplace-level-independent variables regarding PHCs were: size, setting (urban/rural), and governance (federally/privately run). Phlebotomy staff-level independent variables were: age, sex, occupation, years of employment at site and phlebotomy frequency.

### Participants and settings

Swedish primary health care, provided at PHCs, is defined as the first level of health care, and managed at the regional level, i.e., by county councils. According to the Swedish health and medical care policy, every county council must provide residents with good-quality health services and medical care and work toward promoting good health in the entire population. The majority of the PHCs are owned and run by the county councils, and to ensure the quality at privately run PHCs, contracts with the county councils are required [31]. All Swedish PHCs have the same assignment and are organized similarly with the same professions employed. Hence, all PHCs in this study, regardless of governance, had similar working conditions.

In Sweden there is no specific VBSC staff, and VBSC is performed by several personnel categories, including registered nurses (RN), enrolled nurses (EN) (also called assistant, practical, or licensed-to-practice nurses), clinical chemistry laboratory staff and, more rarely, by physicians and other healthcare personnel. Enrolled nurse education is two or three years of secondary school, whereas the nursing program for registered nurses is three years of university studies.

To ensure model robustness regarding cluster (workplace) analyses, only data from PHCs with a minimum of five respondents were included. Therefore, we assessed data from staff (RN and EN) (*n* = 164) at 25 PHCs performing VBSC and on duty during the study period (November 2006–January 2007) in two counties in northern Sweden. All PHCs had similar working conditions and used the same national VBSC practice recommendations [32]. Primary healthcare centre characteristics are summarized in Table 1. Participant characteristics, and guideline adherence regarding outcome variables are summarized in Table 2.

**Table 1:** Primary healthcare centre characteristics

Variable		n (%)
PHC’s location in urban/rural setting		
	Urban^a^	12 (48)
	Rural^a^	13 (52)
Size of PHC		
	Small (<20 employees)	6 (24)
	Medium (20–34 employees)	11 (44)
	Large (>34 employees)	8 (32)
Governance		
	Federally run	23 (92)
	Privately run	2 (8)

^a^: Defined by the Swedish National Rural Development Agency (2007)

Urban = settings with >3000 inhabitants

Rural = settings with <3000 inhabitants

**Table 2:** Demographic characteristics of participants, and frequency of adherence to guidelines in outcome variables

		Values in n (%)				
			1	2	3	4
			Always ask patient to state name and civic number	Never neglect asking for ID with the reason“known”	Always compare pat ID with ID on test request	Always make sure test request and test tube label ID numbers are consistent
		Total (*n* = 164)	Adherence to guideline – within background variable group			
Sex						
	Female	155 (95)	79 (53)	56 (38)	122 (80)	89 (59)
	Male	9 (5)	4 (44)	2 (25)	5 (56)	5 (62)
Occupation						
	Enrolled nurses	64 (39)	37 (59)	27 (44)	52 (84)	41 (65)
	Registered nurses	100 (61)	46 (49)	31 (33)	75 (76)	53 (55)
Employed at worksite						
	<5 years	53 (34)	33 (62)	27 (51)	45 (87)	28 (55)
	5-15 years	52 (34)	24 (48)	16 (32)	42 (82)	32 (64)
	>15 years	49 (32)	22 (45)	11 (24)	34 (69)	30 (63)
Participants’ workplace size						
	Small (<20 empl)	38 (23)	11 (31)	4 (11)	27 (73)	15 (44)
	Medium (20–34 empl)	70 (43)	37 (56)	26 (41)	51 (75)	46 (67)
	Large (>34 empl)	56 (34)	35 (64)	28 (52)	49 (88)	33 (59)
Participants’ workplace setting						
	Urban	78 (48)	47 (63)	36 (50)	61 (80)	43 (56)
	Rural	86 (52)	36 (44)	22 (27)	66 (78)	51 (62)
Governance						
	Federally run	154 (94)	9 (95)	56 (39)	120 (80)	89 (59)
	Privately run	10 (6)	4 (40)	2 (20)	7 (70)	5 (56)
VBSC frequency						
	Every workday	89 (56)	47 (54)	9 (35)	72 (83)	56 (65)
	Every week or less	70 (44)	35 (51)	28 (40)	52 (75)	36 (53)
Total adherence			83(53)	58(38)	127(79)	94(59)
			Missing 7	Missing 10	Missing 3	Missing 5

*- Defined by the Swedish National Rural Development Agency (2007)

Urban setting = >3000 inhabitants

Rural setting = <3000 inhabitants

VBSC = venous blood specimen collection

### Data collection

Data were collected using a self-reported venous blood sampling questionnaire (VBSQ), developed within the project, showing acceptable face and content validity [33, 34] and reliability [33]. The instrument consists of questions on background characteristics (sex, date of birth, occupation, and workplace) and questions on adherence to guidelines based on VBSC procedures as recommended by The Handbook for Healthcare [32], which are almost identical to those in the international Clinical and Laboratory Standards Institute’s H3-A6 VBSC guideline [35] and available online to all phlebotomy staff. In this study, only questions regarding patient ID and handling of test requests were used. It is noteworthy that it was pointed out clearly that respondents were to state how they usually performed VBSC, not if they knew how it should be performed correctly. Participants responded to questions and statements on a 4-point ordinal scale: *never, seldom, often,* or *always*.

Prior to statistical analysis, ordinal data were dichotomized into *correct procedure* (1) and *incorrect procedure* (0), with only one alternative out of four considered to be correct. Outcome variables were: *Always ask patient to state name and civic number* (item 1), *Never neglect asking for ID with the reason “known”* (item 2), *Always compare patient ID with ID on test request* (item 3), and *Always make sure test request and test tube label ID numbers are consistent* (item 4). The independent variable *size of PHC* was categorized according to the total number of employees at site using quartile 1 (Q1) and quartile 3 (Q3) measures. PHCs with a total staff number from minimum to under Q1 (19 employees or less), was categorized as *small* Q1 to under Q3 (20–34 employees) were categorized as *medium sized* and Q3 or higher (35 employees, or more) *large.* In this article we used the definition proposed by the Swedish National Rural Development Agency in which townships with 3000 or more inhabitants are defined as ‘urban’ and smaller communities as ‘rural’ [36], and according to this definition the included PHCs were located in both urban and rural areas (Table 1).

### Ethical considerations

Ethical approval was obtained from the Regional Ethical Review Board prior to data collection (D-No 06–104 M). All participants received written information on the study, as well as the information that participation was not mandatory. Participating in the study was considered accepted informed consent.

### Data analysis

To quantify and assess the variation between different workplaces (PHCs) for *reporting in accordance to guidelines* (1 = yes, 0 = no) we used multilevel logistic analyses, because our material is organized into data from individuals (at a lower level) who are nested into contextual units (clusters), which in our study are the workplaces (at a higher level). Three models were created for each item to apply to our data. The full model contained both workplace (PHC size, urban/rural setting, and governance) and individual (age, sex, occupation, years of employment at site, and VBSC frequency) characteristics. The empty model contained estimates only for the PHC-level random intercept of adherence with VBSC guidelines and was intended to act as a baseline for comparison with the full and adjusted models that take into account both fixed variables and random effect terms. The adjusted model was created in a manual stepwise backward elimination procedure starting with the full model and deleting variables one at a time until only significant variables were left in the model. Evaluation of random effects for the different models were made using intra-class correlation coefficient (ICC) and median odds ratio (MOR). The ICC represents the percentage of the total variation in *reporting in accordance to guidelines* that is accounted for by the cluster (workplace) level and was calculated according to the latent variable method [37]. The purpose of the MOR [38, 39] is to translate the cluster (workplace) level variance into the commonly used odds ratio (OR) scale, which is easily interpreted. The MOR in this study is defined as the median value of the odds ratio between a workplace (PHC) at the highest probability of adherence and a workplace at the lowest probability of adherence. Thus, the MOR shows the extent to which the phlebotomist’s probability of adherence to VBSC guidelines is determined by workplace affiliation. Because the MOR and the ICC are both functions of the cluster level variance they are closely related.

**Fig. 1:**
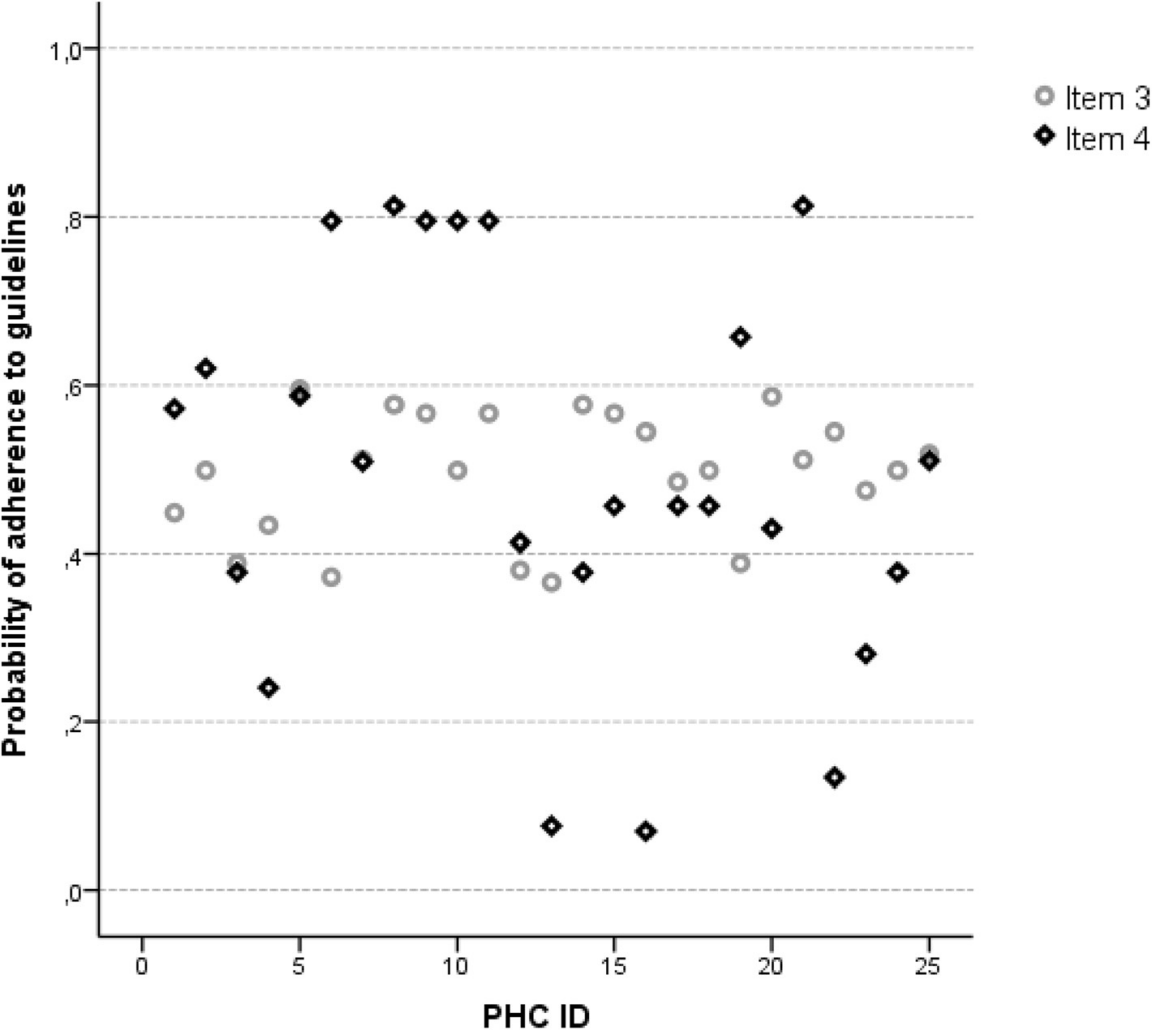
Probability of guideline adherence for an item with high MOR and ICC, and an item with low MOR and ICC. Each point represents one PHC. PHC ID: primary healthcare centre Identification number

To investigate the impact of individual and workplace characteristics on the outcome *reporting in accordance to guidelines* (1 = yes, 0 = no), odds ratios (ORs) and their corresponding 95 % confidence intervals (CIs) from the logistic regression analyses were used. SPSS (IBM SPSS Statistics 20, IBM, New York, US) was used to assess descriptive statistics and to produce Fig. 1. The multilevel logistic regression models were estimated with *R statistics* (version 3.0.2) using the R-package ‘*eha’.*

## Results

The majority of the PHCs were of medium size, and federally run (Table 1). The study participants ranged in age from 24 to 66 years (mean 49.1, SD 9.34), were mostly women (95 %), and working at medium-sized PHCs in both urban and rural settings (Table 2).

Table 2 shows proportions of adherence across independent background variables. A higher proportion of adherence to guidelines was shown for females (item 1–3), for ENs (item 1–4), for shorter time of employment at workplace (item 1–3), for participants working at larger sized PHCs (item 1–3), at urban located PHCs (item1–3), and at federally run PHCs (item 1–4). Divergent results were found regarding participants’ phlebotomy frequency.

Measures of workplace variance in adherence to VBSC guidelines obtained by multilevel logistic regression analyses are shown in Table 3. The results from the empty models show that workplace affiliation significantly explained 36 % to 41 % of the total variation between workplaces in self-reported adherence to three out of four selected guidelines practices (item 1, 2 and 4)*.* Patient ID procedures (items 1 and 2, Table 3) show high MOR (4.06 and 3.66 respectively) in the empty model, with corresponding ICCs indicating that workplace affiliation explains about 40 % and 36 % of the total variance in VBSC adherence between workplaces. Workplace impact decreases to some extent when also controlling for individual, and PHC characteristics in the full and adjusted models. Test request handling procedures (item 3 and 4, Table 3) show diverging results regarding workplace affiliation impact on VBSC practice. When workplace variance is high (empty model, item 4 MOR = 4.21, ICC = 41 %), the probability of adherence to guidelines varies remarkably (probability 0.1–0.8) between different workplaces. In comparison, when workplace variance is non-significant and low (empty model, item 3, MOR = 1.74, ICC = 9 %), the probability of guideline adherence in different workplaces is more similar (probability around 0.5) (Fig. 1). When also controlling for individual and PHC characteristics in the full model, workplace impact decreased to a minimum and therefore had practically no influence for item 3. In the adjusted model for item 4, no fixed variables were significantly associated with the outcome. However, in the full model, workplace impact decreased slightly.

**Table 3:** Measures of association between participant characteristics and primary healthcare centre characteristics and the outcomes (adherence to guidelines) in primary healthcare centres in two counties in northern Sweden, 2007, obtained from analyses

Measures of variation PHC	Always ask patient to state name and civic number	Never neglect asking for ID with the reason “known”	Always compare pat ID with ID on test request	Always make sure test request and test tube label ID numbers are consistent
Empty model^a^				
Sd (SE), *p*	**1.47** (**0.41), <0.001**	**1.36** (**0.41), <0.001**	0.58 (0.37), 0.15	**1.51** (**0.42) <0.001**
MOR	**4.06**	**3.66**	1.74	**4.21**
ICC	**0.40**	**0.36**	0.09	**0.41**
Full model^b^				
Sd (SE), *p*	**1.40** (**0.41), <0.001**	**1.25** (**0.44), 0.004**	0.000034 (3.36), 0.5	**1.40** (**0.42), <0.001**
MOR	**3.81**	**3.30**	1.000	**3.80**
ICC	**0.37**	**0.32**	3.5*10^−10^	**0.37**
Adjusted model^c^				
Sd (SE), *p*	**1.31** (**0.38), <0.001**	**1.22** (**0.41), 0.002**	0.69 (0.42), 0.14	**1.51** (**0.42) <0.001**
MOR	**3.49**	**3.20**	1.93	**4.21**
ICC	**0.34**	**0.31**	0.13	**0.41**
Measures of association – adjusted model OR (95 % CI)				
Age				
Sex				
Male			1	
Female			**5.42** (**1.12-26.20)**	
Occupation				
Enrolled nurse				
Registered nurse				
Employed at worksite (years)				
>15		1	1	
5-15		**1.39** (**0.44-4.36)**	2.27 (0.80-6.39)	
<5		**4.66** (**1.41-15.39)**	**3.35** (**1.09-10.23)**	
PHC setting				
Urban				
Rural				
PHC size (tot # staff)				
Small, <20	1	1		
Medium, 20-34	5.06 (0.87-29.47)	**9.87** (**1.36-71.86)**		
Large, >34	**9.32** (**1.35-64.30)**	**28.36** (**3.06-262.75)**		
VBSC frequency				
Every day		1		
≤ Every week		**2.76** (**1.04-7.29)**		
Governance				
County council				
Private				

Significant values (*p* < .05) in bold characters

Empty model^a^: solely random intercept of adherence with VBSC guidelines

Full model^b^: random intercept of adherence with VBSC guidelines in combination with age, sex, occupation, employed at worksite, PHC setting, PHC size, VBSC frequency and governance

Adjusted model^c^: random intercept of adherence with VBSC guidelines in combination with remaining significant variables after stepwise backward elimination procedure

*MOR* median odds ratio, *CI* confidence interval, *OR* odds ratio, *ID* identification, *Urban/Rural* defined by the Swedish National Rural Development Agency (2007)

Table 3 also shows parameter estimates for the final adjusted model also taking workplace variation into consideration. The analyses showed few significant associations (ORs and corresponding 95 % CIs) between individual and/or PHC characteristics and adherence to guidelines. Women were more likely to adhere with the procedure of comparing patient ID and ID on test request (item 3) than men. Staff employed shorter time on site were more likely to adhere with the procedure of “not neglecting to ask for ID with reason ‘known’”(item 2) and “always compare patient ID with ID on test request (item 3) compared with those employed longer. Staff at medium and large PHCs were more likely to adhere with the procedure of “always ask patient to state name and civic number (item 1) and “never neglect asking for ID with reason ‘known’” (item 2) than staff at small PHCs. Finally, staff performing VBSC every week or less often were more likely to adhere with the procedure of “never neglect asking for ID with reason ‘known’” (item 2) compared with staff who performed VBSC on a daily basis.

## Discussion

This study contributes to knowledge about the relationship between VBSC practice adherence and workplace affiliation. It also identifies both workplace and individual characteristics associated with adherence to VBSC guidelines.

Our data showed differences in self-reported VBSC practices between workplaces. The results of the empty model multilevel analyses demonstrate the impact of PHC affiliation on three out of four items (item 1, 2 and 4). Workplace affiliation impact for these items remained approximately stable (high MOR) in both the full and adjusted models, indicating that workplace affiliation has a considerable impact on patient ID and test request handling procedures, even if controlled for individual and workplace characteristics. This is in line with other studies that shows patient ID procedures [40] and other practices to be strongly associated with the social context of the nurses’ work group [41]. It is known that individuals in a specific group, such as co-workers, who spend a substantial amount of time together undergo processes in which members tend to develop a shared policy and degree of acceptance [42]. The primary healthcare staff in our study could have adopted such practices regarding patient identification practices, as the stable high MOR values showed that the participants followed workplace practices rather than adhering to guidelines [43]. This explanation is plausible for our results, as most of the staff (66 %) had been employed at least 5 years, and therefore had had plenty of time to develop their own sets of prevailing truths regarding VBSC procedures.

Occupational or professional socialization (the acquisition of cultural knowledge and awareness of roles) includes adjustment to new surroundings and learning the behaviors, attitudes and skills necessary to function as a member of a new work organization [44, 45]. Studies of this phenomenon mainly reflect the adaptation every newcomer experiences in their first weeks or months at a new position, ‘realizing and redefining role expectations’ [46]*.* Our study included staff at specific workplaces regardless of employment time. Still, professional socialization processes were probably involved in the associations between workplace affiliation and reported adherence to VBSC guideline practice, since it is very likely that a person employed at a workplace displays at least some practices similar to their peers.

Phlebotomists at medium and large PHCs were more likely to adhere to guidelines regarding patient ID (item 1 and 2), in contrast to findings by Jacobs et al. who found that large settings were more bureaucratic and therefore more likely to have barriers against best practice [11]. Staff who work closely together on a daily basis have more opportunities to talk about procedures and may substitute checking guidelines with conference with their peers, which in turn contribute to ‘shared basic assumptions’ [47]. Furthermore, the probability of a phlebotomist to encounter the same patient frequently is more likely at small PHCs than at larger ones. Staff who meet with patients on numerous occasions might eventually recognize them (‘known patient’ factor), and therefore for example identify the patient by asking for passive agreement “Your birthdate is June 5^th^ 1977, right?”, a non-acceptable hazardous procedure [48]. Thus, staff might finally remember the patients’ names and even their civic numbers, and gradually neglect the guidelines for correct ID practice, which is in line with our findings of the low proportions of adherence regarding both item 1 (53 %) and item 2 (38 %). Such procedures might eventually jeopardizes patient safety, a fact which can be crucial for certain patient groups who have their blood analyzed frequently, and therefore pay regular visits to the PHC. Furthermore, both fewer years of employment at site, larger PHCs, and less VBSC frequency were associated with better adherence. However, regarding the correct procedure of always ensure coherence between patient ID and information on test request (item 3), the vast majority (79 %) of the participants reported practices in accordance with guidelines. These findings are somewhat contradictory, since the assurance of coherence depends on the fact that the patient in advance has stated name and civic number. Thus, our overall results indicate that the low proportion reporting in line with patient ID guidelines stems from an increasing negligence in terms of adherence to guidelines which in turn may contribute to an increased incidence of the ‘known patient’ factor [49].

Our findings support the hypothesis that contextual factors, such as a workplace, influence staff who spend a substantial period of time at site, and the fact that they tend to develop practices that to a large extent can be explained by workplace affiliation. Unsatisfactory low proportions of guideline adherence (38-79 %) reported by our participants. Who should be held responsible for suboptimal adherence to VBSC practice guidelines at PHCs; the workplace or the individual phlebotomists? Our data reveal flaws at both levels. Recent studies on CPG adherence have mainly focused on the organizational aspect. Studies to identify reasons for individual hazard behavior that might explain habitual choices to ignore important safety rules are few. To explain the origins of errors within healthcare, the pendulum of accountability, having swung fully towards the organization, is now swinging back towards the individual [50]. It is crucial to balance organizational and individual factors to ensure the best possible conditions for a just culture [51] that promotes safe care and balances between a no-blame culture with no individual accountability, and a culture in which staff are blamed for all errors and near misses. Further research combining both organizational and individual factors is warranted contributing to higher levels of CPG adherence and increased patient safety.

## Limitations

In surveys performed as cross sectional studies it is difficult to determine what is dependent on what. However in this study the focus was to explore to what extent workplace affiliation explains variation of self-reported adherence to VBSC practices. For this reason we assume that typical problems in cross-sectional studies are not obvious in this study. All PHCs included were situated in two counties in northern Sweden, and data were collected in 2007. Therefore, the results may not be generalized to conditions at present PHCs, or at PHCs in other areas. The low number of included phlebotomy staff at some PHCs (cluster level) may have influenced the result. The low number of included privately run PHCs (two) and males (nine included) makes interpretation precarious. Our survey did not include questions on personal aspects, such as attitudes towards guidelines, which limited the interpretation of the individual phlebotomist’s impact on adherence to CPGs.

## Conclusion

Workplace affiliation largely explains variances in self-reported adherence to VBSC patient ID and test request handling guidelines practices among PHC phlebotomy staff. Primary healthcare centre factors, as well as individual phlebotomist factors, could be barriers contributing to poor levels of guideline practice adherence. Healthcare managers should therefore take both organizational and individual factors into consideration when planning interventions aimed to enhance guideline practice adherence among healthcare staff. Furthermore, additional attention and support are required for staff who are found to not to adhere to CPGs. Further research is warranted regarding the association between specific risk factors and guideline adherence to provide healthcare managers with knowledge in order to enable tailored interventions to ascertain patient safety.

## Abbreviations

CPG: clinical practice guidelines
ICC: intra-class correlation
ID: identification
MOR: median odds ratio
OR: odds ratio
PHC: primary healthcare centre
VBSC: venous blood specimen collection
